# Supplementary material for: How covariate control can bias our insights into brain architecture and pathology
Source: Sci Rep. 2026 Apr 17;16:17804. doi: 10.1038/s41598-026-47122-4 (PMC13247238; doi:10.1038/s41598-026-47122-4)
Supplement: Supplementary file 1 — Supplementary Material 1 [file 41598_2026_47122_MOESM1_ESM.pdf]

Supplementary

Basic concept of the simulation approach in Experiment 3

The basic concept of the simulation design used in Experiment 3 is as follows: firstly, lesion masks are generated in a real data sample of stroke patients. These indicate the lesioned area in each patient. Secondly, the researcher defines a ground truth that explains how a fictional, simulated deficit is caused by neural damage. For example, one could decide that any lesion to the supramarginal gyrus causes a deficit. Such a rule is applied to simulate a score for each patient based on each lesion map. Thirdly, the neural correlates of the simulated score are mapped onto the brain with a lesion-deficit inference method. Lastly, the precision of the statistical map is evaluated. In the above-mentioned example, valid lesion-deficit inference should identify the supramarginal gyrus as the neural correlate of the deficit, and any systematic deviation uncovers the limitations of the inference method.

Supplementary Table 1: Descriptive statistics

IQR – interquartile range; SD – standard deviation.

	All participants, N = 183
Age, years mean (SD; range)	65.8 (14.8; 26-98)
Sex Female/Male, %	42.1/57.9
Lesion size in cm^3, median(IQR; range)	2.7 (0.8; 13.4; 0.1-251.5)
Lesioned hemisphere L/R/both, N	66/103/14
Hypertension, %	67.8
Visuoconstructive ability score, mean (SD; range)	9.1 (1.8; 2-11)
Selective attention, number of omissions, mean (SD; range)	4.7 (6.6; 0-33)
Short-term memory, maximal correctly repeated digit span, mean (SD; range)	6.0 (1.7; 2-11)

Supplementary Table 2: Statistics Experiment 3 – Condition 1

Detailed results of statistical analyses in experiment 3 for the first condition where only an impact of lesion location was simulated. Statistical comparison was performed by Wilcoxon Signed-rank tests. Reported p-values were corrected by the Bonferroni algorithm to account for multiple comparisons across 4 covariates. Asterisks indicate significance at * p<0.05; ** p<0.01; *** p<0.001. IQR – interquartile range.

Covariate	Median(IQR)	Statistical comparison to no control condition
Total number of significant voxels		
No control	548 [128; 1697]	-
Age	540 [149;1613]	Z=-0.75, p=1
Sex	540 [112; 1637]	Z=-1.86, p=0.25
Hypertension	653 [123; 1598]	Z=-0.48, p=1
Lesion Volume	11 [4; 98]	Z=-4.70, p< **0.0001*** **
Positive predictive value (=Sensitivity)		
No control	0.06 [0.02; 0.22]	-
Age	0.06 [0.02; 0.22]	Z=0.95, p=1
Sex	0.06 [0.02; 0.21]	Z=0.50, p=1
Hypertension	0.06 [0.02; 0.21]	Z=1.04, p=1
Lesion Volume	0.04 [0.00; 0.17]	Z=1.43, p=0.61
Number of false negatives		
No control	307 [218; 396]	-
Age	295 [220; 395]	Z=-0.42, p=1
Sex	325 [215; 398]	Z=-1.17, p=0.97
Hypertension	320 [212; 394]	Z=0.02, p=1
Lesion Volume	375 [243; 481]	Z=-4.54, p< **0.0001*** **
Dice similarity coefficient		
No control	0.07 [0.02; 0.16]	-
Age	0.07 [0.02; 0.15]	Z=0.50, p=1
Sex	0.07 [0.02; 0.12]	Z=0.98, p=0.98
Hypertension	0.07 [0.03; 0.12]	Z=0.79, p=1
Lesion Volume	0.00 [0.00; 0.02]	Z=4.28, p< **0.0001*** **

Supplementary Table 3: Statistics Experiment 3 – Condition 2

Detailed results of statistical analyses in experiment 3 for the second condition where both an impact of lesion location and the covariate were simulated. Statistical comparison was performed by Wilcoxon Signed-rank tests. Reported p-values were corrected by the Bonferroni algorithm to account for multiple comparisons across 3 covariates. Asterisks indicate significance at * p<0.05; ** p<0.01; *** p<0.001.

Covariate	Baseline without covariate control Median(IQR)	With covariate control Median(IQR)	Statistical comparison
Total number of significant voxels			
Age	55 [11;298]	484 [27; 1818]	*Z*=-4.62, *p*< **0.0001*** **
Sex	223 [14; 1473]	411 [45; 1850]	*Z*=-4.62, *p*< **0.0001*** **
Hypertension	198 [56; 506]	307 [75; 724]	*Z*=-3.94, *p*< **0.0001*** **
Positive predictive value (=Sensitivity)			
Age	0.03 [0.01; 0.08]	0.04 [0.02; 0.12]	*Z*=-0.86, *p*=1
Sex	0.05 [0.01; 0.12]	0.07 [0.03; 0.12]	*Z*=-0.26, *p*=1
Hypertension	0.04 [0.00; 0.09]	0.03 [0.00; 0.11]	*Z*=0.99, *p*=0.97
Number of false negatives			
Age	366 [224; 491]	351 [191; 427]	*Z*=3.61, *p*= **0.0009*** **
Sex	324 [190; 439]	304 [201; 426]	*Z*=3.49, *p*= **0.0014*** **
Hypertension	348 [230; 445]	322 [214; 444]	*Z*=2.69, *p*= **0.0215***
Dice similarity coefficient			
Age	0.01 [0.00; 0.05]	0.05 [0.00; 0.08]	*Z*=-2.59, *p*= **0.029***
Sex	0.02 [0.02; 0.09]	0.04 [0.02; 0.08]	*Z*=-2.17, *p*= 0.08
Hypertension	0.02 [0.00; 0.09]	0.02 [0.00; 0.09]	*Z*=-1.34, *p*= 0.54

Supplementary Analysis Experiment 3 with lesion volume

In the second condition of Experiment 3, we used a simulation approach to investigate the effect of covariate control in a setting where causal relationships in the data are known. We simulated a deficit based on the lesion load to a randomly chosen brain region and a covariate, hence causal relationships were known by design. For the main analyses, we decided to omit the covariate lesion volume from this condition, because the causal relationship between lesion volume and lesion location is already complex and ambiguous. In other words, this covariate may strongly violate the methodological setting that we tried to enforce by the simulation.

We still applied the simulation design with a causal impact of both the lesion load and the covariate on lesion volume as a supplementary analysis. We followed the strategy

from the main analyses. First, we reused the 30 randomly placed spheres from the main analyses as ground truth regions. Scores of 0±1 were simulated for patients with damage to the ground truth region, and else 1.5±1. To include a direct effect of lesion volume, we computed the z-standardisation and subtracted z(lesion volume)*0.5 points from the score, i.e., higher lesion volume led to a more pathological performance. Like in the main analyses, outcome variables were total number of significant voxels, positive predictive value, and number of false negatives.

Results

Results and statistics are shown in Supplementary Table 4. The first thing that stood out was that this simulation—in contrast to the main analyses reported in Supplementary Table 3—already found far-reaching statistically significant results in the baseline condition, with a median of ~12,800 voxels. After controlling for lesion volume, these results were far less extensive (median ~11 voxels), although this did not lead to significant differences in the sensitivity of the results. However, control for lesion volume significantly increased the number of false alarms.

In summary, control for lesion volume did not improve the results in this condition. However, the simulation, although following the same strategy as the main analyses with the other covariates, created outstandingly widespread results without control, but very limited results with control. These findings indicate that the simulation condition may be more complex than assumed, reflecting our concerns that led us to exclude this analysis from the main results.

Supplementary Table 4: Statistics Supplementary Analysis Experiment 3 – Condition 2 with lesion volume

Detailed results of statistical analyses in the supplementar analysis to experiment 3 for the variable lesion volume. Statistical comparison was performed by Wilcoxon Signed-rank tests. Reported p-values were corrected by the Bonferroni algorithm to account for multiple comparisons across 3 covariates. Asterisks indicate significance at * p<0.05; ** p<0.01; *** p<0.001.

Covariate	Baseline without covariate control Median(IQR)	With covariate control Median(IQR)	Statistical comparison
Total number of significant voxels			
Lesion Volume	12787 [9025;14662]	11.5 [4; 39]	Z=4.78, p< **0.0001*** **
Positive predictive value (=Sensitivity)			
Lesion Volume	0.02 [0.02; 0.03]	0.04 [0; 0.2]	Z=-1.83, p=0.20
Number of false negatives			
Lesion Volume	73 [19; 164]	375.5 [243; 481]	Z=-4.78, p< **0.0001*** **
Dice similarity coefficient			
Lesion Volume	0.04 [0.03; 0.06]	0.00 [0.00; 0.02]	Z=3.82, p< **0.001*** **
